# Expression and Function of the Homeostatic Molecule Del-1 in Endothelial Cells and the Periodontal Tissue

**DOI:** 10.1155/2013/617809

**Published:** 2013-12-12

**Authors:** Jieun Shin, Kavita B. Hosur, Kalyani Pyaram, Ravi Jotwani, Shuang Liang, Triantafyllos Chavakis, George Hajishengallis

**Affiliations:** ^1^Department of Microbiology, School of Dental Medicine, University of Pennsylvania, 240 South 40th Street, Philadelphia, PA 19104, USA; ^2^Department of Microbiology and Immunology, University of Michigan, Ann Arbor, MI 48109, USA; ^3^Oral Health and Systemic Disease Research Group, University of Louisville School of Dentistry, Louisville, KY 40292, USA; ^4^Division of Vascular Inflammation, Diabetes and Kidney, Department of Medicine, Technical University Dresden, 01307 Dresden, Germany; ^5^Department of Clinical Pathobiochemistry and Institute for Clinical Chemistry and Laboratory Medicine, Technical University Dresden, 01307 Dresden, Germany

## Abstract

Developmental endothelial locus-1 (Del-1) is an endothelial cell-secreted protein that limits the recruitment of neutrophils by antagonizing the interaction between the LFA-1 integrin on neutrophils and the intercellular adhesion molecule (ICAM)-1 on endothelial cells. Mice with genetic or age-associated Del-1 deficiency exhibit increased neutrophil infiltration in the periodontium resulting in inflammatory bone loss. Here we investigated additional novel mechanisms whereby Del-1 could interfere with neutrophil recruitment and inflammation. Treatment of human endothelial cells with Del-1 did not affect the expression of endothelial molecules involved in the leukocyte adhesion cascade (ICAM-1, VCAM-1, and E-selectin). Moreover, genetic or age-associated Del-1 deficiency did not significantly alter the expression of these adhesion molecules in the murine periodontium, further ruling out altered adhesion molecule expression as a mechanism whereby Del-1 regulates leukocyte recruitment. Strikingly, Del-1 inhibited ICAM-1-dependent chemokine release (CXCL2, CCL3) by neutrophils. Therefore, Del-1 could potentially suppress the amplification of inflammatory cell recruitment mediated through chemokine release by infiltrating neutrophils. Interestingly, Del-1 was itself regulated by inflammatory stimuli, which generally exerted opposite effects on adhesion molecule expression. The reciprocal regulation between Del-1 and inflammation may contribute to optimally balance the protective and the potentially harmful effects of inflammatory cell recruitment.

## 1. Introduction

Developmental endothelial locus-1 (Del-1) is an endothelial cell-secreted 52 kDa protein, originally described for its role in embryonic vascular development [1]. The Del-1 molecule consists of three epidermal growth factor- (EGF-) like repeats at the N-terminus (E1, E2, E3) followed by two discoidin I-like domains at the C-terminus (C1, C2) [1], hence also known as EGF-like repeats and discoidin I-like domains 3 (EDIL3). More recently, Del-1 was identified as a novel antagonist of the leukocyte LFA-1 integrin (CD11a/CD18) [2, 3]. Specifically, Del-1 inhibits the interaction of LFA-1 with intercellular adhesion molecule (ICAM)-1 on endothelial cells and thereby blocks the firm adhesion of leukocytes onto the vascular endothelium [2]. Since firm adhesion is a prerequisite for transendothelial migration [3, 4], Del-1 ultimately limits the migration of human neutrophils through endothelial cells [5].

Using animal models of periodontitis [5], a prevalent chronic inflammatory disease that leads to the destruction of tooth-supporting tissues (periodontium) and is often associated with systemic complications [6–9], we have recently established a homeostatic role for Del-1 in local inflammation [5]. Specifically, Del-1 regulates neutrophil recruitment to the periodontium and inhibits inflammation and bone loss [5]. This concept was based on work with distinct *in vivo* models. We first showed that Del-1 expression is diminished in the periodontium of old mice relative to young mice, correlating with excessive neutrophil recruitment and IL-17A-dependent inflammatory bone loss in old mice [5]. Second, we demonstrated that young Del-1-deficient (*Edil*3^−/−^) mice spontaneously exhibit heavy neutrophil infiltration and inflammatory bone loss that were dependent on the LFA-1 integrin and the IL-17 receptor [5]. Moreover, we observed an inverse relationship between Del-1 expression and IL-17A expression in human gingival biopsy samples, with Del-1 dominating in healthy gingiva and IL-17A in inflamed gingiva [5]. This is consistent with reciprocal regulation between the two molecules. Indeed, the periodontal tissue production of IL-17A is inhibited by Del-1, whereas the expression of Del-1 in endothelial cells is in turn inhibited by IL-17A [5]. The ability of Del-1 to act as a gatekeeper of leukocyte recruitment and inflammation is also supported by a subsequent study in a different model involving inflammation-mediated adrenal gland dysfunction [10]. Another mechanism by which Del-1 could suppress inflammation is through its ability to promote the clearance of platelet-derived microvesicles by the endothelium [11].

In the present work, we investigated additional mechanisms that could contribute to the capacity of Del-1 to limit the recruitment of neutrophils. Moreover, we obtained a more comprehensive understanding of the factors that can influence Del-1 expression in endothelial cells. Our findings show that whereas Del-1 does not influence the expression of endothelial molecules involved in the leukocyte adhesion and transmigration cascade (ICAM-1, VCAM-1, and E-selectin), Del-1 inhibits ICAM-1-dependent activation of neutrophils and, specifically, ICAM-1-dependent chemokine induction by neutrophils. Moreover, only a subset of the inflammatory stimuli that enhance ICAM-1 and VCAM-1 expression also regulated the expression of Del-1, although generally in a converse manner to the regulation of ICAM-1 and VCAM-1. Therefore, it appears that Del-1 both regulates and is regulated by inflammation and this dynamic interplay likely maintains a balance between the protective and the potentially destructive effects of inflammatory cell recruitment.

## 2. Materials and Methods

### 2.1. Reagents

*Porphyromonas gingivalis *ATCC 33277 was grown anaerobically from frozen stocks on modified Gifu anaerobic medium (GAM) based blood agar plates for 5-6 days at 37°C, followed by anaerobic subculturing for 18–24 hours at 37°C in modified GAM broth (Nissui Pharmaceutical). Pam_3_CSK_4_ lipopeptide and *Escherichia coli* lipopolysaccharide (LPS) were purchased from InVivogen. TNF, IFN*γ*, IL-17A, and IL-17F were obtained from R&D Systems and serum amyloid A from Abcam. Human Del-1, ICAM-1 fused to the Fc fragment of IgG (ICAM-1-Fc), and Fc protein control were purchased from R&D Systems, whereas Del-1-Fc was prepared in our laboratory as follows. The cDNA for human *Edil3* (encoding Del-1) was obtained from OriGene. The gene encoding the full-length Del-1 protein sequence was amplified by PCR followed by cloning into the HindIII and KpnI sites of the mammalian expression vector pSecTag2 (InVitrogen), which contains an *N*-terminal secretory tag (murine Igk leader sequence) and a *C*-terminal polyhistidine tag (His-tag). The human IgG Fc gene (synthesized by Integrated DNA Technologies) was cloned between *Edil3* and His-tag at EcoRI and XhoI sites and in frame with both sequences. Del-1 was therefore expressed as a soluble Fc-fusion protein (Del-1-Fc; 81.3 kDa) secreted into the culture medium of transfected HEK-293F suspension cells (InVitrogen). The protein was purified by Ni-affinity chromatography after loading concentrated culture supernatants onto a His-Trap column (GE Healthcare) connected to an ÄKTA-FPLC system (GE Healthcare). The eluted Del-1-Fc was pooled and dialyzed, and the identity and purity of the protein were confirmed using immunoblotting (with anti-Del-1 and anti-Fc antibodies from R&D Systems and Southern Biotech, resp.) and SDS-PAGE.

### 2.2. Mice and Bone Loss Measurements

All animal procedures were approved by the Institutional Animal Care and Use Committee, in compliance with established federal and state policies. The generation of C57BL/6 *Edil*3^−/−^ mice has been described [2]. In experiments of aging, *Edil*3^−/−^ mice and their *Edil*3^+/+^ wild-type (WT) littermates were reared in parallel under specific-pathogen-free conditions. Bone loss in these mice was assessed using a morphometric method as previously described [5] using a Nikon SMZ800 microscope (Nikon Instruments) and a 40 × objective. Images of the maxillae were captured using a Nicon Digital Sight DS-U3 camera controller (Nikon Instruments) and bone heights were measured using NIS-Elements software (Nikon Instruments).

### 2.3. Immunofluorescence Histochemistry

Maxillae with intact surrounding tissue were fixed in 4% paraformaldehyde, decalcified in Immunocal solution (Decal) for 15 days, and embedded in OCT compound. Serial mesiodistal sections parallel to the long axis of the teeth (sagittal) were stained using monoclonal antibodies to ICAM-1 (YN1/1.7.4; Abnova), VCAM-1 (MVCAM. A [429]; BioLegend), or with polyclonal antibodies to CD31 or E-selectin (both from LSBio), followed by secondary reagents (AlexaFluor488- or AlexaFluor594-conjugated goat anti-rabbit IgG; Molecular Probes). The specificity of staining was confirmed by using appropriate isotype controls or normal rabbit IgG followed by AlexaFluor488- or AlexaFluor594-conjugated goat anti-rabbit IgG. Images were captured using a laser-scanning confocal microscope (Olympus FV1000). Fluorescence intensity was quantified using the Image J software (NIH; http://rsb.info.nih.gov/ij).

### 2.4. Human Umbilical Vein Endothelial Cells (HUVEC)

HUVEC were seeded in 6-well culture plates in complete EBM-2 medium (Lonza) and grown to confluence. The cells were then washed and treated without or with recombinant human Del-1 (R & D Systems) at 1 or 5 *μ*g/mL. After 2-hour incubation in a humidified atmosphere at 37°C and 5% CO_2_, the cells were collected by trypsinization and total RNA was isolated for real-time PCR analysis as described below.

### 2.5. HL-60 Cells

The human promyelocytic leukemia HL-60 cell line was obtained from the American Type Culture Collection (ATCC CCL-240) and was cultured at 37°C and 5% CO_2_, in RPMI 1640 supplemented with 10% fetal bovine serum (ATCC), 100 U/mL penicillin, and 100 *μ*g/mL streptomycin. HL-60 cells were treated with 1 *μ*M all-*trans* retinoic acid (ATRA; Sigma) for 4 days and their differentiation into neutrophils was monitored by FACS analysis of CD11b expression using phycoerythrin-conjugated anti-human CD11b mAb (BD Pharmingen) on a BD Accuri C6 flow cytometer.

### 2.6. Quantitative Real-Time PCR

Total RNA was extracted from dissected gingival tissue or from cultured HUVEC using the PerfectPure RNA cell kit (5 Prime, Fisher) and quantified by spectrometry at 260 and 280 nm. The RNA was reverse-transcribed using the High-Capacity cDNA Archive kit (Applied Biosystems), and real-time PCR with cDNA was performed using the ABI 7500 Fast System, according to the manufacturer's protocol (Applied Biosystems). TaqMan probes, sense primers, and antisense primers for real-time PCR of genes investigated in this paper were purchased from Applied Biosystems.

### 2.7. ICAM-1-Dependent Chemokine Induction by HL-60 Neutrophils

Ninety-six-well plates were coated with anti-human IgG-Fc (20 *μ*g/mL) followed by ICAM-1-Fc, Del-1-Fc, or human IgG-Fc control (all at 10 *μ*g/mL). ATRA-differentiated HL-60 neutrophils (see above) were seeded on these plates (10^5^ cells/well) and cultured in the absence or presence of soluble Del-1 (1–20 *μ*g/mL). In some experiments, the cells were seeded and cultured directly on 96-well plates; in this case, the cells were stimulated with Pam_3_CSK_4_ or LPS (0.1 *μ*g/mL and 1.0 *μ*g/mL, resp.). In all cases, following incubation for 24 hours, culture supernatants were collected and assayed for CCL3, CXCL2, or TNF by ELISA using kits from R&D Systems, PeproTech, or BD Biosciences, respectively.

### 2.8. Statistical Analysis

Data were evaluated by one-way analysis of variance and the Dunnett multiple-comparison test with the InStat program (GraphPad Software). Where appropriate (comparison of two groups only), two-tailed *t*-tests were done. *P* values of less than 0.05 were considered significant. With the exception of the mouse aging study (Figure 3), all experiments were performed two times or more for verification.

## 3. Results

### 3.1. Expression of Endothelial Cell Adhesion Molecules in Del-1 Deficiency

**Figure 1:**
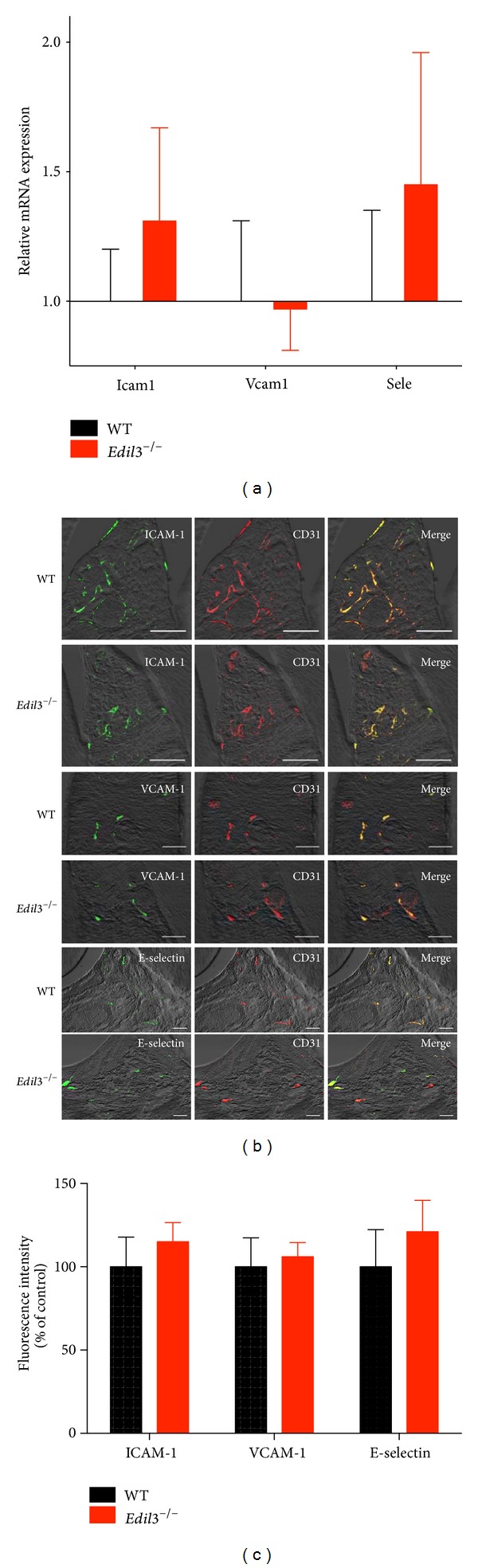
Expression of endothelial adhesion molecules in Del-1 deficiency. (a) Gingiva dissected from 16-week-old C57BL/6 *Edil*3^+/+^ (WT) and *Edil*3^−/−^ mice were processed for quantitative real-time PCR analysis of mRNA expression of the indicated genes; results were normalized to those of GAPDH mRNA and expressed as fold change in *Edil*3^−/−^ transcript levels relative to WT, the average value of which was taken as 1. (b) Sagittal sections of interdental gingiva from 16-week-old C57BL/6 WT or *Edil*3^−/−^ mice were stained for ICAM-1, VCAM-1, or E-selectin and CD31, as indicated. Shown are representative overlays of differential interference contrast and fluorescent confocal images, with colocalization demonstrated in merged images (scale bar, 50 *μ*m). (c) The fluorescence intensities of the images shown here and of additional representative images from independent mice (5 per group) were quantified using ImageJ analysis. In (a) and (c), data are means ± SD (*n* = 5 mice per group). No statistically significant differences were detected between *Edil*3^−/−^ and WT littermate controls.

**Figure 2:**
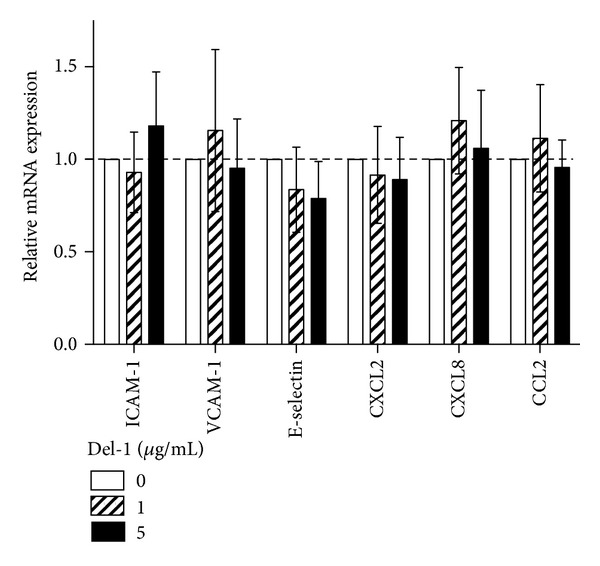
Effect of Del-1 treatment on human endothelial cell expression of adhesion molecules and chemokines. HUVEC were treated with the indicated concentrations of human Del-1 and were assessed for mRNA expression of the indicated cell adhesion and chemokine molecules by quantitative real-time PCR; results were normalized against GAPDH mRNA and expressed as fold change relative to untreated controls, which were assigned an average value of 1. Data are means ± SD (*n* = 5 sets of endothelial cells). No statistically significant differences were detected between Del-1 treatments and untreated controls.

We have previously shown that Del-1 deficiency is associated with increased recruitment of inflammatory cells (predominantly neutrophils) to the periodontium, owing to lack of Del-1-dependent antagonism of LFA-1-mediated extravasation [5]. Moreover, Del-1 deficiency was associated with altered periodontal tissue expression of a number of inflammatory mediators and receptors, such as IL-17A, chemokine C-X-C ligand 2 (CXCL2), and chemokine CXC receptor 2 (CXCR2) [5]. We thus reasoned that possible increased expression of endothelial molecules crucially involved in the leukocyte adhesion and transmigration cascade (ICAM-1, VCAM-1, and E-selectin) could have contributed to the enhanced inflammatory cell infiltration seen in *Edil*3^−/−^ mice. However, quantitative real-time PCR analysis of mRNA expression in gingival tissue did not detect significant differences between *Edil*3^−/−^ mice and WT littermate controls with regard to expression of ICAM-1, VCAM-1, and E-selectin at 16 weeks (Figure 1(a)), an age at which *Edil*3^−/−^ mice exhibit dramatic bone loss relative to WT littermate controls [5]. Using immunofluorescence histochemistry, we confirmed that ICAM-1, VCAM-1, and E-selectin were comparably expressed at the protein level in WT and *Edil*3^−/−^ mice at 16 weeks (Figures 1(b) and 1(c)). All three adhesion molecules colocalized with CD31 (endothelial cell marker) (Figure 1(b)), thus confirming their expression by the gingival endothelium. These findings suggest that altered expression of these major endothelial cell adhesion molecules is unlikely to contribute to the enhanced neutrophil recruitment seen in Del-1 deficiency. Furthermore, Del-1 treatment of human endothelial cells did not affect their expression of ICAM-1, VCAM-1, and E-selectin or of leukocyte-recruiting chemokines (CXCL2, CXCL8, and CCL2) (Figure 2). These data collectively suggest that Del-1 regulates the LFA-1-dependent recruitment of neutrophils possibly without direct effects on endothelial cell inflammatory function.

### 3.2. Expression of Del-1 and Endothelial Cell Adhesion Molecules and Bone Loss as a Function of Age

**Figure 3:**
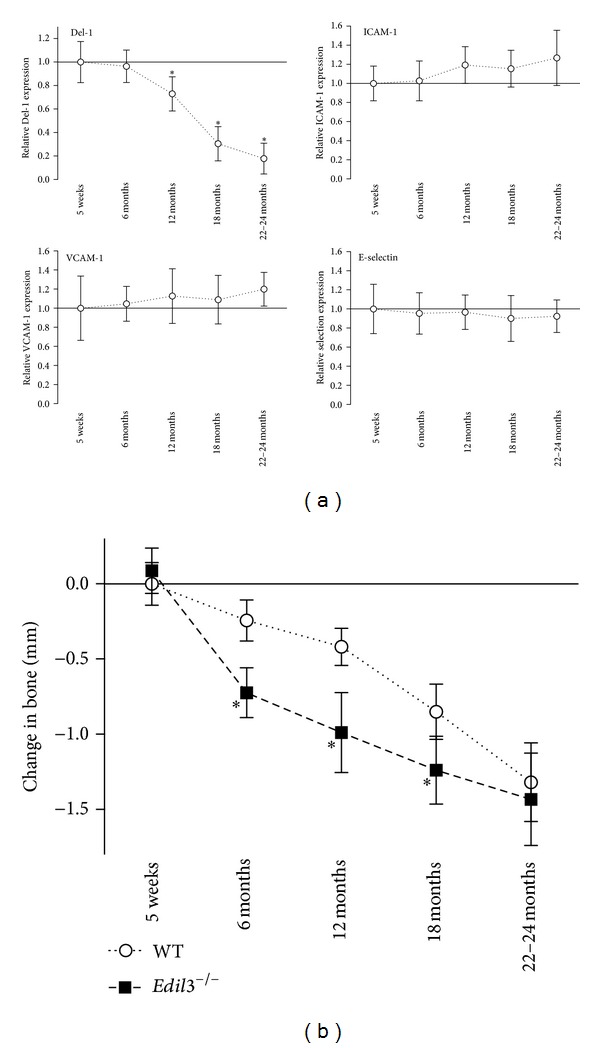
Expression of Del-1 and endothelial cell adhesion molecules and bone loss as a function of age. (a) Gingiva dissected from WT mice at the indicated ages were processed for quantitative real-time PCR analysis of mRNA expression of the indicated genes; results were normalized to those of GAPDH mRNA and expressed as fold change in transcript levels relative to those of 5-week-old WT mice, the average value of which was taken as 1. (b) Time course of naturally occurring bone loss in aging WT mice in comparison to age-matched *Edil*3^−/−^ mice; negative values indicate bone loss relative to bone measurements in 5-week-old wild-type mice. Data are means ± SD (*n* = 6–8 mice per group). *P < 0.01 as compared to 5-week-old WT mice (a) or between *Edil*3^−/−^ and WT mice (b).

We have previously shown that 18-month-old WT mice have decreased expression of Del-1 in the periodontium but increased bone loss as compared to young mice (8–10 weeks of age) [5]. In new experiments in WT mice, we monitored the periodontal expression of Del-1 in parallel with ICAM-1, VCAM-1, and E-selectin from the age of 5 weeks to very old age (up to 24 months) (Figure 3(a)). As expected, the expression of Del-1 significantly declined as a function of age; however, the expression of the three adhesion molecules was not significantly altered (Figure 3(a)). We moreover monitored the age-associated bone loss of the same WT mice in parallel with their *Edil*3^−/−^ littermates. Although *Edil*3^−/−^ mice consistently had more bone loss than WT mice up to the age of 18 months, the two groups exhibited comparable bone loss in very old age (22–24 months) (Figure 3(b)). This age coincided with severe decline (>80%) of Del-1 expression (Figure 3(a)), which renders the 22–24 month old WT mice practically Del-1 deficient. The observation that the periodontal bone loss in aging WT mice correlates with significant changes in the expression of Del-1, but not of ICAM-1, VCAM1, or E-selectin, further supports the notion that the age-associated Del-1 deficiency is an important determinant of age-associated periodontitis.

### 3.3. Del-1 Inhibits ICAM-1-Dependent Chemokine Production by Neutrophils

**Figure 4:**
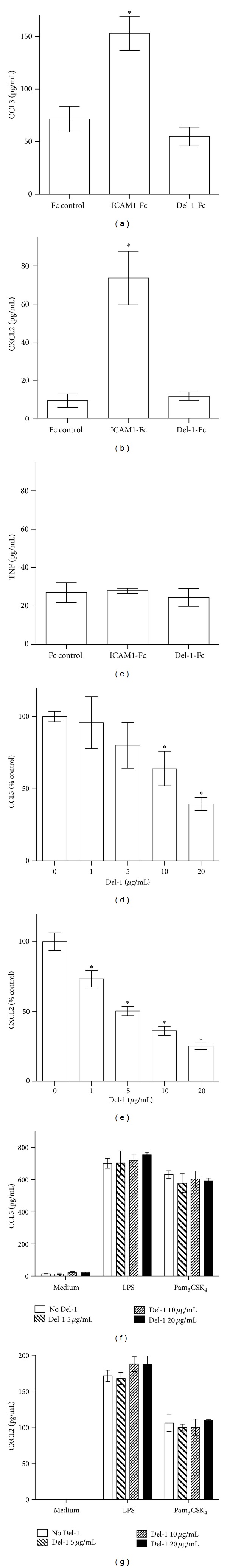
Del-1 inhibits ICAM-1-dependent chemokine production by neutrophils. (a)–(c) ATRA-differentiated HL-60 neutrophils were cultured for 24 hours on plates coated with Fc protein control, ICAM-1-Fc, or Del-1-Fc, and induction of release of CCL3 (a), CXCL2 (b), and TNF (c) was assayed by ELISA. (d)-(e) ATRA-differentiated HL-60 neutrophils were cultured for 24 hours on ICAM-1-Fc-coated plates in the presence of the indicated concentrations of soluble Del-1, and induction of release of CCL3 (d) and CXCL2 (e) was assayed by ELISA. (f)-(g) ATRA-differentiated HL-60 neutrophils were stimulated for 24 hours with 1 *μ*g/mL Pam_3_CSK_4_ or 0.1 *μ*g/mL LPS, in the absence or presence of the indicated concentrations of Del-1, and induction of release of CCL3 (f) and CXCL2 (g) was assayed by ELISA. Data are means ± SD ((a)–(e), *n* ≥ 4 and (f)-(g), *n* = 2 sets of HL-60 cells). *P < 0.01 as compared to Fc control (a)–(c) or to no-treatment (“0” Del-1 concentration).

The interaction of endothelial ICAM-1 with *β*_2_ integrins in monocytes was shown to induce the release of chemokine C-C ligand 3 (CCL3; also known as macrophage inflammatory protein-1*α*) [12]. CCL3 production by monocytes was also induced in monocytes cultured on ICAM-1-coated plates [12]. Using a similar system, we determined whether immobilized ICAM-1 could induce chemokine production in ATRA-differentiated HL-60 neutrophils, and, if so, whether soluble Del-1 could block ICAM-1–dependent chemokine production. In our experiments, ICAM-1 was used as a fusion protein with IgG Fc (ICAM-1-Fc) and was coated on plates treated with anti-Fc antibodies. We showed that ATRA-HL-60 cells cultured on ICAM-1-coated plates indeed released CCL3 (Figure 4(a)) as well as CXCL2 (Figure 4(b)) but not TNF (Figure 4(c)). Although secreted by endothelial cells, Del-1 can also become attached to the endothelial cell surface via specific binding interactions [13, 14]. In the same experiments, therefore, we tested whether Del-1-coated plates (Del-1-Fc coated on anti-Fc-treated plates) could similarly support chemokine responses by HL-60 neutrophils. However, when cultured on Del-1-coated plates, HL-60 neutrophils failed to release CCL3 or CXCL2 at levels significantly higher than the baseline levels determined on Fc protein-coated plates (Figures 4(a), and 4(b), resp.). Importantly, soluble Del-1 added into the HL-60 cell cultures on ICAM-1-coated plates inhibited the release of CCL3 (Figure 4(d)) and CXCL2 (Figure 4(e)) in a dose-dependent manner, although the inhibitory effect on the latter chemokine was more potent. Importantly, Del-1 failed to inhibit LPS- or Pam_3_CSK_4_-induced release of CCL3 (Figure 4(f)) and CXCL2 (Figure 4(g)); hence, Del-1 does not antagonize chemokine release by TLR4 or TLR2 agonists, respectively. This finding supports the specificity of the Del-1 inhibitory effect on chemokine production that was dependent on the ICAM-1-LFA-1 interaction.

### 3.4. Regulation of Del-1, ICAM-1, and VCAM-1 by Inflammatory Stimuli

**Figure 5:**
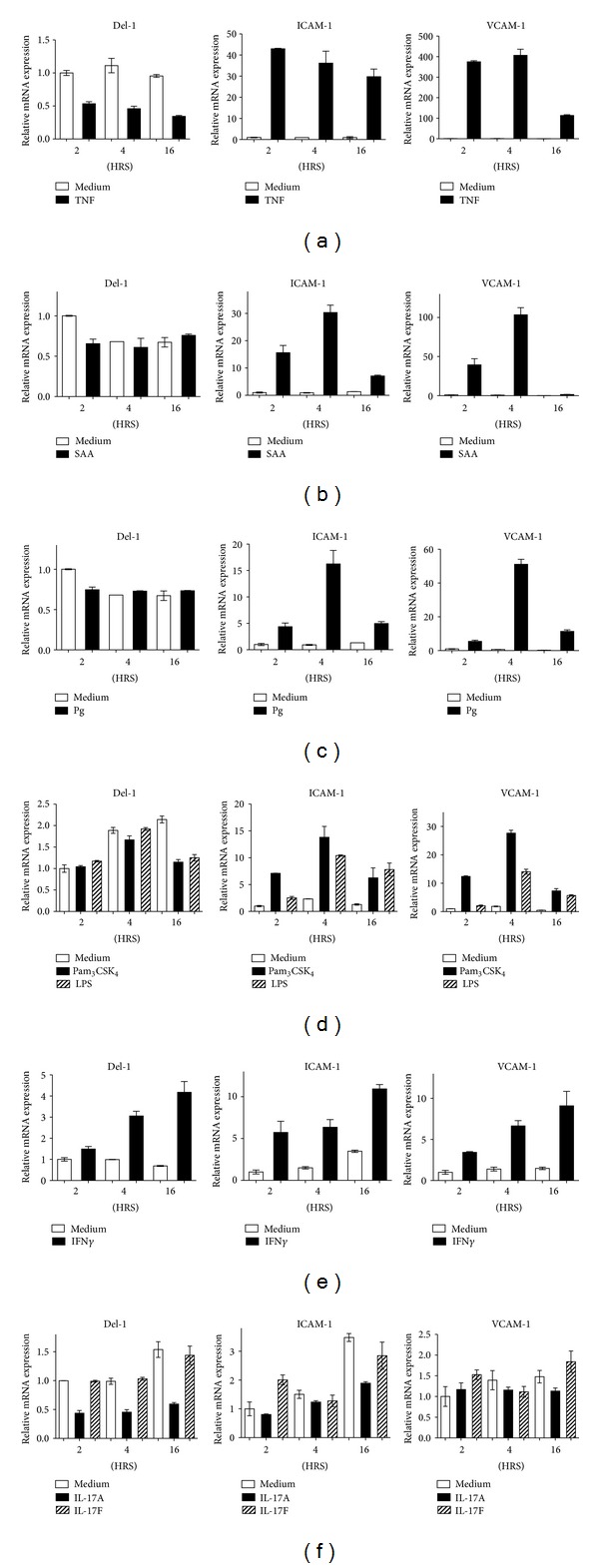
Regulation of Del-1 versus ICAM-1 and VCAM-1 by inflammatory stimuli. HUVEC were cultured for the indicated time intervals with 10 ng/mL TNF (a), 5 *μ*g/mL serum amyloid A (SAA; (b)), *P. gingivalis* (Pg; MOI = 10 : 1) (c), Pam_3_CSK_4_ or LPS (both at 0.5 *μ*g/mL; (d)), 10 ng/mL IFN*γ* (e), or IL-17A or IL-17F (both at 10 ng/mL; (f)) and assayed for Del-1, ICAM-1, and VCAM-1 mRNA expression. Results were normalized to those of GAPDH mRNA and expressed as fold change in transcript levels relative to those of medium-treated cells at 2 hours (HRS), the average value of which was taken as 1. The medium-treated groups in (b) and (c) are the same (SAA and Pg were tested together but were separated in the graphs for enhanced clarity). Data are means ± SD of duplicate determinations from one of three independent sets of experiments that yielded similar results.

Since Del-1 antagonizes the effects of adhesion molecules in terms of inflammatory cell recruitment and induction of chemokines, we hypothesized that inflammatory stimuli might exert differential effects on the regulation of Del-1 expression in HUVEC as compared to that of adhesion molecules. This hypothesis was generally supported by our findings. TNF inhibited Del-1 and strongly upregulated ICAM-1 and VCAM-1 at all three time intervals examined (2, 4, and 16 hours) (Figure 5(a)). Both adhesion molecules were upregulated also by serum amyloid A (Figure 5(b)), the periodontal pathogen *P. gingivalis* [15] (Figure 5(c)), and purified TLR ligands such as Pam_3_CSK_4_ (TLR2) and LPS (TLR4) (Figure 5(d)). On the other hand, these proinflammatory stimuli did not upregulate Del-1 but generally did not inhibit its expression either, except for a single timepoint: serum amyloid A and *P. gingivalis* inhibited Del-1 expression at 2 hours (Figures 5(b) and 5(c), resp.), whereas Pam_3_CSK_4_ and LPS inhibited Del-1 expression at 16 hours (Figure 5(d)). Unexpectedly, IFN*γ* enhanced the expression of both Del-1 and the adhesion molecules at all three timepoints (Figure 5(e)). Consistent with our previous report [5], IL-17A strongly inhibited Del-1 expression (Figure 5(f)). However, the IL-17F isoform failed to influence Del-1 expression (Figure 5(f)). In contrast to TNF, neither IL-17A nor IL-17F exerted a consistent upregulatory effect on ICAM-1 or VCAM-1 expression; in fact, both cytokines had complex effects that included either modest upregulation or inhibition or no effect at all, depending on the timepoint examined (Figure 5(f)).

## 4. Discussion

The process of leukocyte extravasation involves a cascade of low- and high-affinity adhesive interactions with the endothelium lining the vascular bed of the infected or inflamed tissue [3, 4, 16]. Initially, transient rolling interactions between endothelial cell surface molecules, such as E-selectin, and glycoprotein ligands on circulating leukocytes lead to their deceleration followed by their firm adhesion and subsequent crawling on the endothelium, during which leukocytes seek an appropriate site for transmigration. Firm adhesion and crawling are primarily mediated by leukocyte integrins that interact with endothelial counterreceptors such as VCAM-1 and ICAM-1 [3, 4, 16]. In this paper, we showed that endothelial molecules critically involved in the leukocyte adhesion cascade (E-selectin, VCAM-1, and ICAM-1) are comparably expressed in the periodontium of WT or *Edil*3^−/−^ mice. Therefore, we ruled out that altered expression of these major endothelial adhesion molecules could contribute to the increased neutrophil recruitment seen in Del-1 deficiency [5]. Moreover, we now showed that the periodontal tissue expressions of ICAM-1, VCAM-1, and E-selectin do not significantly change with aging, in contrast to Del-1 expression which dramatically declines. Therefore, the ability of Del-1 to antagonize the interaction between the LFA-1 integrin and ICAM-1 (hence, the firm adhesion of neutrophils onto the vascular endothelium) [2] remains the most likely mechanism for the increased neutrophil infiltration seen in genetic or age-associated Del-1 deficiency [5].

Our data indicated that TNF can act in more ways than IL-17 to “prime” the endothelial cells for increased leukocyte adhesion as it could both upregulate adhesion molecule expression and inhibit Del-1 expression, whereas IL-17 consistently mediated only the latter function. Considering that Del-1 antagonizes LFA-1-dependent leukocyte adhesion, our data might explain in part why IL-17-expressing T cells (Th17), but not Th1 cells, are crucially dependent on the LFA-1 integrin for their recruitment [17].

The leukocyte-endothelial cell interactions during the leukocyte adhesion cascade not only represent a mechanism for leukocyte transmigration to peripheral tissues but also participate in intracellular signaling in leukocytes that result in ICAM-1-dependent chemokine production [12]. This mechanism could contribute in sustaining continuous recruitment of leukocytes by augmenting the chemotactic signals generated during the inflammatory responses. Intriguingly, although Del-1 does not seem to directly regulate the activity of neutrophils [5], it suppressed ICAM-1-dependent, but not TLR-dependent, chemokine release by neutrophils. These novel findings suggest that Del-1 could additionally interfere with the ability of transmigrating neutrophils to amplify inflammatory cell recruitment via the release of CXCL2 and CCL3. In this regard, CXCL2 is chemotactic for neutrophils, whereas CCL3 induces the migration of monocytes and lymphocytes [16, 18].

Most of the inflammatory stimuli tested failed to upregulate Del-1 (they either inhibited Del-1 expression or had no effect). This is consistent with the need for rapid recruitment of leukocytes to sites of infection or inflammation, which would require minimal or no inhibition of LFA-1-dependent extravasation by Del-1. It was, therefore, surprising that IFN*γ* upregulated the expression of Del-1. On the other hand, however, IFN*γ* was shown to also elicit anti-inflammatory effects, such as inhibition of Th17 differentiation [19] or of osteoclastogenesis [20]. It could thus be speculated that the ability of IFN*γ* to upregulate Del-1 expression is relevant within the context of its regulatory functions, although further research is warranted to determine the biological significance of the IFN*γ*-induced Del-1 expression.

The capacity of Del-1 to both regulate and be regulated by inflammation (IL-17, TNF) [5] (and this study) indicates a dynamic regulatory interplay that ostensibly acts to optimally balance the protective and the potentially harmful effects of inflammatory cell recruitment. In this regard, although inflammation-induced downregulation of Del-1 would be desirable in the acute response to infection, it would conversely become detrimental in a chronic setting. In this regard, the age-associated decline of Del-1 expression in the periodontium and the concomitant unaltered expression of proinflammatory endothelial adhesion molecules, as shown in this study, represents a mechanism of homeostatic breakdown that contributes to periodontitis.

In summary, our current data in the context of our previous reports [2, 5] suggest that Del-1 inhibits local inflammation in tissues that express it, such as the periodontium, by competing with the ICAM-1-dependent firm adhesion and transmigration of neutrophils, as well as with their capacity for ICAM-1-dependent chemokine release that could amplify the recruitment of inflammatory cells. Altered expression of endothelial molecules involved in the leukocyte adhesion cascade was ruled out as an additional mechanism for the increased neutrophil recruitment in the Del-1-deficient periodontium of *Edil*3^−/−^ mice or old WT mice. Del-1 can thus provide a promising platform for developing novel therapeutic approaches for periodontitis and other inflammatory diseases.
